# Supplementary material for: Preparation of Spherical Cellulose Nanocrystals from Microcrystalline Cellulose by Mixed Acid Hydrolysis with Different Pretreatment Routes
Source: Int J Mol Sci. 2022 Sep 15;23(18):10764. doi: 10.3390/ijms231810764 (PMC9502823; doi:10.3390/ijms231810764)
Supplement: Supplementary file 1 [file ijms-23-10764-s001.zip › ijms-1878136-supplementary.pdf]

Supplementary Materials:

Preparation of spherical cellulose nanocrystals from microcrystalline cellulose by mixed acid hydrolysis with different pretreatment routes

Peng Zhu [1,*], Luyao Feng [1], Zejun Ding [1] and Xuechun Bai [1]

[1] College of Textile Science and Engineering (International Institute of Silk), Zhejiang Sci-Tech University, Hangzhou 310018, China

* Correspondence: zhupeng@zstu.edu.cn

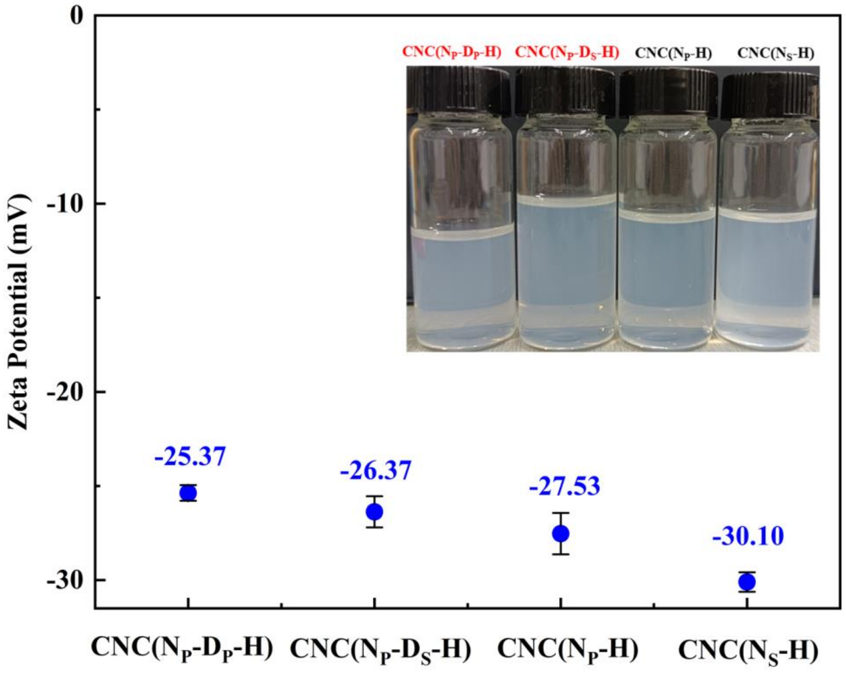

Figure S1. Zeta potential of obtained spherical CNC suspensions with different pretreatments and their optical images after storage for 8 months in the upper right corner

A dispersion stability experiment was carried out by observing spherical CNC suspensions with different pretreatments prepared 8 months ago. From the optical image in the upper right corner (Figure S1), it can be seen that spherical CNC suspensions are well dispersed and stable after storage for 8 months. In addition, the zeta potential test was performed on zeta potential analyzer (Zetasizer Nano S, UK).

Zeta potential is an important indicator to characterize the stability of the dispersion system. Generally, the higher the absolute value of the zeta potential, the greater the electrostatic repulsion between the particles and the better the physical stability. The absolute values of the zeta potential from spherical CNC suspensions with different pretreatments are all higher than 25, meaning that they all have good dispersion stability, which is consistent with the dispersion stability experiment.

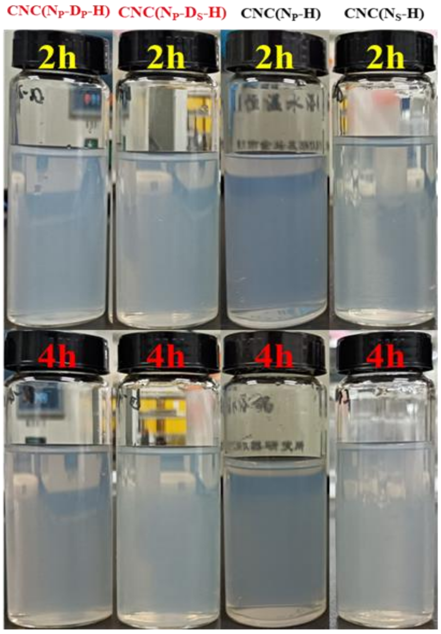

Figure S2. Suspensions of obtained spherical CNC with different hydrolysis time

As shown in Figure S2, when the hydrolysis time is 4h, the color of the suspension turns grey which is presumed to be carbonization. So the hydrolysis time in this work was fixed at 2 h to avoid carbonization caused by further hydrolysis.
